# Efficacy of Remifentanil in Patients Undergoing Cardiac Surgery: A Systematic Review and Network Meta-Analysis

**DOI:** 10.7759/cureus.51278

**Published:** 2023-12-29

**Authors:** Hiromu Okano, Yuki Kataoka, Masaaki Sakuraya, Yoshitaka Aoki, Hiroshi Okamoto, Eriya Imai, Tsutomu Yamazaki

**Affiliations:** 1 Department of Critical Care Medicine, St. Luke's International Hospital, Tokyo, JPN; 2 Department of Social Medical Sciences, Graduate School of Medicine, International University of Health and Welfare, Tokyo, JPN; 3 Section of Clinical Epidemiology, Department of Community Medicine, Kyoto University Graduate School of Medicine, Kyoto, JPN; 4 Department of Healthcare Epidemiology, Kyoto University Graduate School of Medicine/School of Public Health, Kyoto, JPN; 5 Department of Systematic Reviewers, Scientific Research Works Peer Support Group (SRWS-PSG), Osaka, JPN; 6 Department of Internal Medicine, Kyoto Min-iren Asukai Hospital, Kyoto, JPN; 7 Department of Emergency and Intensive Care Medicine, JA Hiroshima General Hospital, Hiroshima, JPN; 8 Department of Anesthesiology and Intensive Care, Hamamatsu University School of Medicine, Hamamatsu, JPN; 9 Division of Anesthesiology, Mitsui Memorial Hospital, Tokyo, JPN

**Keywords:** adult cardiac surgery, critical care anesthesiology, postoperative mechanical ventilation, opioid use, remifentanil

## Abstract

Remifentanil, characterized by its ultra-short action duration and nonorgan-dependent metabolism, is applied in postcardiac surgery settings worldwide. While previous studies have compared its efficacy with that of other opioids, it has never been compared to a single specific opioid. Here, we evaluated whether remifentanil shortens mechanical ventilation (MV) times in patients after cardiac surgery. We identified randomized controlled trials that compared various opioids in adults (≥18 years) admitted to the intensive care unit after cardiac surgery. The primary outcome was the duration of MV, expressed as the mean difference (MD) in minutes, with a 95% confidence interval (CI). A 60-min reduction was considered significant based on prior research. Data were sourced from MEDLINE, the Cochrane Central Register of Controlled Trials, EMBASE, the World Health Organization International Clinical Trials Platforms Search Portal, and ClinicalTrials.gov, and a frequentist network meta-analysis was conducted. The eight identified studies indicate no differences in the duration of MV between remifentanil and fentanyl (MD 0.09 min; 95%CI -36.89-37.08), morphine (MD -19 min; 95%CI -55.86-16.21), or sufentanil (MD -2.44 min; 95%CI -67.52-62.55). Our study revealed that remifentanil did not reduce MV times in patients after cardiac surgery. The study protocol was registered with the Open Science Forum (https://osf.io/) (DOI 10.17605/OSF.IO/YAHW2).

## Introduction and background

Adequate pain management in the intensive care unit (ICU) is critical to improve patient outcomes and reduce the duration of mechanical ventilation (MV) [1]. Opioids, such as morphine, fentanyl, sufentanil, alfentanil, and remifentanil, are frequently used for this purpose. However, improper administration of these opioids can lead to adverse effects including respiratory depression and extended ICU stays [2]. Remifentanil is distinct from other opioids because of its rapid onset and offset of action, along with its unique pharmacokinetics, rendering it a potentially valuable option in critical care settings [3]. Despite these advantages, existing studies have yielded mixed results on its effectiveness. Previous meta-analyses often identified a high level of data heterogeneity and findings were thus inconclusive [4-6]. Moreover, the most current guidelines [7], which recommend remifentanil over other opioids for postoperative cardiac care, are not directly transferable to healthcare systems where, like in Japan, the choice of opioids is limited to remifentanil, fentanyl, and morphine [8,9].

Given this gap in the existing literature, in this review, we conducted a network meta-analysis (NMA) to compare the benefits and risks of remifentanil with those of other opioids, particularly in postoperative cardiac patients. Our objective was to develop a more comprehensive understanding of the role of remifentanil in the management of ICU pain.

## Review

Materials and methods

Protocol and Registration

This systematic review was designed according to the Preferred Reporting Items for Systematic Reviews and Meta-Analyses 2020 (PRISMA-2020) and PRISMA for Network Meta-Analyses (PRISMA-NMA) [10]. We registered the study protocol with the Open Science Forum (https://osf.io/) (DOI 10.17605/OSF.IO/YAHW2) (Additional file 1).

Eligibility Criteria

Type of studies: Randomized controlled trials (RCTs) that assessed remifentanil levels after cardiac surgery were included. We did not apply language or country restrictions and included all available papers, including published and unpublished articles, conference abstracts, and letters. We did not exclude studies based on the observation period or publication year.

Study Participants

The inclusion criteria consisted of adult patients who were 18 years of age or above. These patients were required to be mechanically ventilated in ICU settings. Additionally, they should have undergone either emergency, urgent, or elective cardiac surgery. Another key inclusion factor was their admission to the ICU where they were receiving opioids such as remifentanil, fentanyl, morphine, sufentanil, and alfentanil. As for the exclusion criteria, we specifically omitted patients who had undergone endovascular surgery.

Types of Outcomes

Primary outcome: The primary outcome of interest in our study was the duration of mechanical ventilation (MV). This is quantified as the time span period starting from the conclusion of cardiac surgery to the point when the patient is weaned off the ventilator. We also treated the time to extubation in a similar manner, considering it as the endpoint of mechanical ventilation.

Secondary outcomes: The secondary outcomes included a couple of key measures. The first was the ICU length of stay, which we measured from the completion of cardiac surgery until the patient's discharge from the ICU, with the duration recorded in minutes. Based on prior research, we identified a clinically significant reduction in the MV period as being 60 minutes. The second secondary outcome revolved around adverse events (AEs). These were defined according to the criteria set by the original authors of the studies we included. They encompassed a range of issues, including the incidence rates of nausea and vomiting, hemodynamic instability, and delirium.

Search Method

The following electronic bibliographic databases were searched: MEDLINE via PubMed (Additional file 2, Appendix 1), the Cochrane Central Register of Controlled Trials (CENTRAL) in the Cochrane Library (Additional file 2, Appendix 2), and EMBASE (Additional file 2, Appendix 3). We also searched the World Health Organization International Clinical Trials Platforms Search Portal (ICTRP) (Online Resource 2, Appendix 4) and ClinicalTrials.gov (Additional file 2, Appendix 5) for ongoing trials. No language restrictions were imposed. Each search query included the following terms: “remifentanil,” fentanyl”, ”sufentanil, “alfentanil, and “opioid”. The literature search was performed on June 14, 2023.

We also checked the reference lists of such studies, including international guidelines by the American Society of Critical Care Medicine [11] and the Pan-American and Iberian Federation of Critical Care and Intensive Care Medicine Societies [7] as well as the reference lists of eligible studies and articles citing eligible studies. We asked the authors of the original studies to provide unpublished or additional data. Citation searches were conducted using citationchaser (https://estech.shinyapps.io/citationchaser/).

Data collection and analysis

Selection of the Studies

Two independent reviewers (HO and EI) screened the titles and abstracts of identified studies and assessed their eligibility based on the full texts. We contacted the original authors if relevant data were missing. Disagreements between the two reviewers were resolved by discussion; if this failed, a third reviewer (MS) acted as an arbiter.

Data Extraction and Management

Two reviewers (HO and EI) independently extracted data from the included studies using a standardized data collection form. The form included information on the study design, study population, interventions, and outcomes. Any disagreements were resolved by discussion; if this failed, a third reviewer (MS) acted as an arbiter.

Network Meta-Analysis

Group-level data were entered into the analysis. We used binomial likelihood for dichotomous outcomes and normal likelihood for continuous outcomes. We synthesized the study effect sizes using a random-effects NMA model and accounted for correlations induced by multigroup designs using multivariate distributions. The variance in the random-effects distribution (heterogeneity variance) was used to measure across-study and within-comparison variability in the treatment effects. The analysis was implemented using MetaInsight (https://crsu.shinyapps.io/metainsightc/) [12].

Assessment of Reporting Bias

We also performed an extensive search for unpublished trials on the Clinical Trial Registry System (ClinicalTrials.gov and ICTRP). To assess the outcome reporting bias, we compared the outcomes defined in the trial protocols with those reported in the resulting publications.

Assessment of the Transitivity Assumption

The potential effect modifiers were age and type of surgery. We checked whether these variables were similarly distributed across the study's drug by comparison.

Assessment of Confidence for Each Outcome

Two reviewers (HO and EI) evaluated the confidence for each primary outcome using the CINeMA tool (https://www.cinema-tools.com/) [13]. The CINeMA framework includes the following domains: within- and across-study bias, indirectness, imprecision, heterogeneity, and incoherence. For within‐study bias and indirectness, CINeMA calculates the contribution of each study in each network estimate and combines these contributions with the study‐specific evaluations (low, moderate, high) to rate the relative effect for each comparison in the network. The domains of imprecision, heterogeneity, and incoherence use a prespecified clinically important size of the effect to specify the margin of clinical equivalence between two interventions.

Subgroup and Sensitivity Analysis

We conducted a subgroup analysis excluding patients who were co-administered sedatives. To assess the robustness of our primary findings, we performed a sensitivity analysis by excluding studies deemed to have a high risk of bias in the primary outcomes. We also analyzed participants who had completed their respective studies using complete datasets.

Protocol Deviations

We had initially planned to execute a sensitivity analysis by excluding studies incorporating imputed statistics. However, because no such studies were found, this particular analysis was not performed. Moreover, the original protocol considered mortality at hospital discharge; however, this outcome measure was not reported in any of the included studies and could therefore not be extracted. Also, the protocol originally used time to use ventilation as an outcome, but time to extubation was evaluated as equivalent and integrated. Therefore, we performed a sensitivity analysis that excluded studies that used time extubation as an outcome.

Results

Study Selection

**Figure 1:**
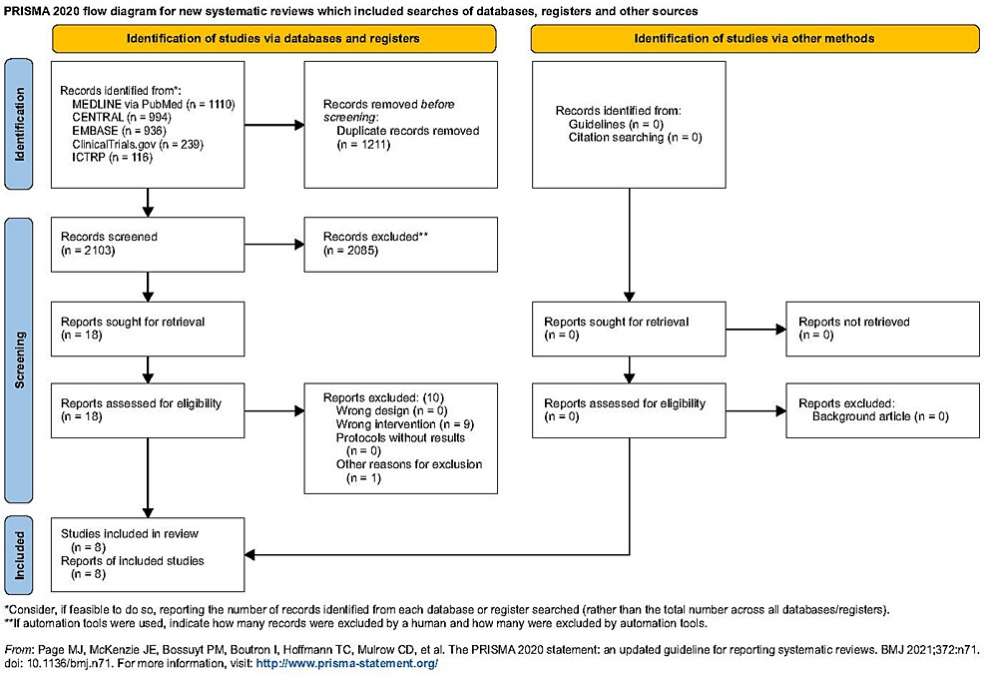
Flow diagram of study inclusion CENTRAL, Cochrane Central Register of Controlled Trials; ICTRP: International Clinical Trials Platforms Search Portal

We identified 2,103 studies, including eight RCTs (691 participants) that were eligible for inclusion (Figure 1) (Table 1) [14-21].

**Table 1:** List of reports excluded from this review and reasons for exclusion

Reason for exclusion	References
Wrong intervention	Rasmussen LA, Ryhammer PK, Greisen J, Bhavsar RR, Lorentzen AG, Jakobsen CJ. Ultrashort-acting remifentanil is not superior to long-acting sufentanil in preserving cognitive function-a randomized study. Journal of clinical anesthesia. 2016;33:127-34. doi: 10.1016/j.jclinane.2016.03.023.
Wrong intervention	Oztekin DS, Oztekin I, Issever H, Göksel O, Cinar B, Canik S. Postoperative effects of opioid analgesics administered via continuous perfusion and patient-controlled analgesia after open heart surgery. Yakugaku Zasshi. 2006;126(7):499-504. doi: 10.1248/yakushi.126.499.
Wrong intervention	Myles PS, Hunt JO, Fletcher H, Watts J, Bain D, Silvers A, Buckland MR. Remifentanil, fentanyl, and cardiac surgery: a double-blinded, randomized, controlled trial of costs and outcomes. Anesthesia and analgesia. 2002;95(4):805-12, table of contents. doi: 10.1097/00000539-200210000-00004.
Wrong intervention	Maddali MM, Kurian E, Fahr J. Extubation time, hemodynamic stability, and postoperative pain control in patients undergoing coronary artery bypass surgery: an evaluation of fentanyl, remifentanil, and nonsteroidal anti-inflammatory drugs with propofol for perioperative and postoperative management. Journal of clinical anesthesia. 2006;18(8):605-10. doi:
Wrong intervention	Khanykin B, Siddiqi R, Jensen PF, Bigler DR, Atroshchenko GV. Comparison of remifentanil and low-dose fentanyl for fast-track cardiac anesthesia: a prospective randomized study. The heart surgery forum. 2013;16(6):E324-8. doi: 10.1532/hsf98.2013229.
Wrong intervention	Guggenberger H, Schroeder TH, Vonthein R, Dieterich HJ, Shernan SK, Eltzschig HK. Remifentanil or sufentanil for coronary surgery: comparison of postoperative respiratory impairment. Eur J Anaesthesiol. 2006;23(10):832-40. doi: 10.1017/s0265021506000251.
Wrong intervention	Engoren M, Luther G, Fenn-Buderer N. A comparison of fentanyl, sufentanil, and remifentanil for fast-track cardiac anesthesia. Anesthesia and analgesia. 2001;93(4):859-64. doi: 10.1097/00000539-200110000-00011.
Wrong intervention	Bhavsar R, Ryhammer PK, Greisen J, Rasmussen LA, Jakobsen CJ. Remifentanil Compared With Sufentanil Does Not Enhance Fast-Track Possibilities in Cardiac Surgery-A Randomized Study. J Cardiothorac Vasc Anesth. 2016;30(5):1212-20. doi: 10.1053/j.jvca.2015.12.021.
Wrong intervention	Alavi SM, Kish RF, Farsad F, Imani F, Sheikhvatan M. Intravenous sufentanil and morphine for post-cardiac surgery pain relief using patient-controlled analgesia (pca) device: a randomized double-blind clinical trial. Pakistan Journal of Medical Sciences. 2010;26(1):137-41.
Other reason for exclusion	D. Loncar Stojiljkovic, M. P. Stojiljkovic.Efficacy and safety of postoperative use of morphine, fentanyl and remifentanyl after coronary artery bypass grafting.40th International Symposium on Intensive Care & Emergency Medicine 2021. Critical Care. 2021;25(1):383. doi: 10.1186/s13054-021-03769-1.

These eight studies can be categorized as follows: a comparative study of remifentanil and fentanyl [17], an evaluation of sufentanil compared to remifentanil [14], a comparison between morphine and remifentanil [16], an assessment of morphine versus sufentanil [18], a study investigating the differences between morphine and fentanyl [15], two studies assessing the effects of alfentanil in relation to morphine [20,21], and a comparative study involving three treatment groups (remifentanil, fentanyl, and morphine) [19].

**Figure 2:**
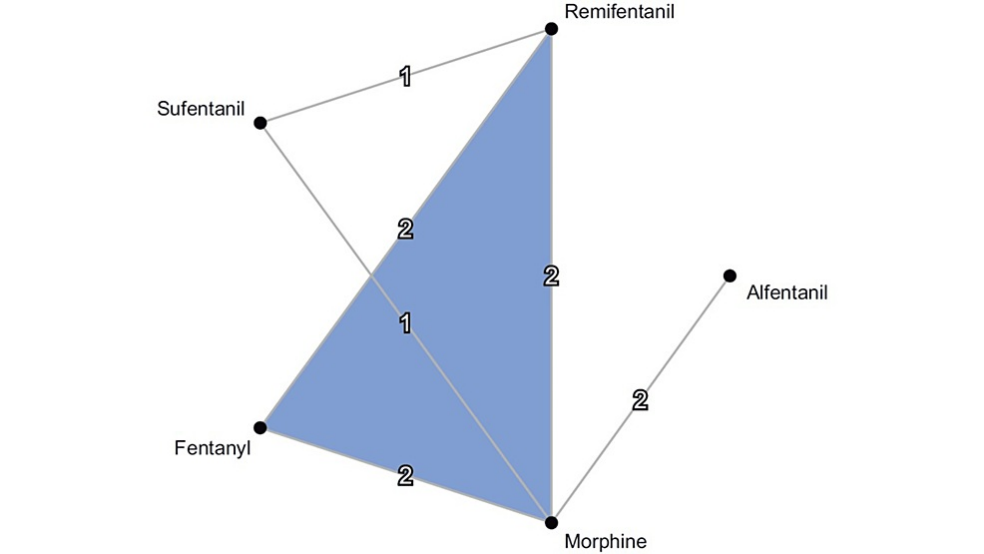
Network plot of studies comparing opioids after cardiac surgery When RCTs for direct comparisons were available, they are depicted by connections between the nodes. RCTs: Randomized controlled trials

Figure 2 presents the primary outcomes of this review. However, since some of the data were inadequate, we could not generate a network diagram for the secondary outcomes.

Study Characteristics and Risk of Bias Within Studies

Table 2 shows the participants, interventions, comparisons, outcomes, and cohort characteristics of the included studies. All eight studies [14-21] reported the MV duration and four studies [14-17] reported the ICU length of stay. Five studies reported AEs [14,16,17,19,20]. Table 3 depicts the risk of bias.

**Table 2:** Characteristics of included studies

Authors	Year	Total number of patients randomized	Types of Surgery	Interventions	Comparisons	Age(Interventions/Comparisons)	Outcomes
Mcmenemin et al. [21]	1988	40	Coronary artery bypass surgery	Morphine	Alfentanil	56(46-66) / 57(46-67)	Duration of mechanical ventilation
Checketts et al. [20]	1998	105	Scheduled for elective cardiac bypass surgery	Morphine	Alfentanil	61(38-74) / 58(39-79)	Time to extubation Adverse Event
Gurbet et al. [19]	2004	75	Off-pump coronary artery bypass surgery﻿	Morphine, Fentanyl	﻿Remifentanil	59.0 ±1.8 / 60.5 ± 2.3/ 58.2 ± 2.6	Duration of mechanical ventilation Adverse Event
Bastin et al. [18]	2005	20	Scheduled for cardiac surgery	Morphine	Sufentanil	63 (3) / 63 ( 4)	Duration of mechanical ventilation
Muellejans et al. [17]	2006	80	﻿Elective coronary artery and/or heart valve surgery	Fentanyl and Midazolam	﻿Remifentanil and Propofol	66.5 ± 7.0 / 65 ± 8.1	Duration of mechanical ventilation Duration of ICU stay Adverse Event
Baltali et al. [16]	2009	58	Scheduled for elective cardiac bypass surgery	Morphine	﻿Remifentanil	58 ±8 / 57±8	Duration of mechanical ventilation Duration of ICU stay Adverse Event
Oliver et al. [15]	2011	145	Scheduled for elective cardiac surgery	Morphine and Propofol	Fentanyl and Propofol	63 (53-72) / 62 (55-71)	duration of mechanical ventilation duration of ICU stay
Alavi et al. [14]	2014	249	Coronary artery bypass grafting	Sufentanil	﻿Remifentanil	56.8±13.30/56.2±13.92	Duration of mechanical ventilation Duration of ICU stay Adverse Event
Age was stated as Mean ±SD or Median (1IQR-3IQR) or Mean (SEM).							

**Table 3:** Summary of the risk of bias for the duration of mechanical ventilation

Authors	Bias arising from the randomization process	Bias due to deviations from intended interventions	Bias due to missing outcome data	Bias in measurement of the outcome	Bias in selection of the reported result	Overall risk of bias
Mcmenemin 1988 [21]	Low Risk	Low Risk	Low Risk	Low Risk	Some Concerns	Some Concerns
Cheketts 1998 [20]	Low Risk	Low Risk	Low Risk	Low Risk	Some Concerns	Some Concerns
Gurbet 2004 [19]	Some Concerns	Low Risk	Low Risk	Low Risk	Some Concerns	Some Concerns
Bastin 2005 [18]	Some Concerns	Low Risk	Low Risk	Low Risk	Some Concerns	Some Concerns
Muellejans 2006 [17]	Low Risk	Low Risk	﻿High Risk^a^	Low Risk	Some Concerns	High Risk
Baltali 2009 [16]	Low Risk	Some Concerns	Low Risk	Low Risk	Some Concerns	Some Concerns
Oliver 2011 [15]	Low Risk	Low Risk	Low Risk	Low Risk	Some Concerns	Some Concerns
Alavi 2014 [14]	High Risk^b^	Some Concerns	Low Risk	Low Risk	Some Concerns	High Risk
a. Outcomes are missing 10%. b. No description of the allocation method						

Primary Outcome: Duration of MV

**Figure 3:**
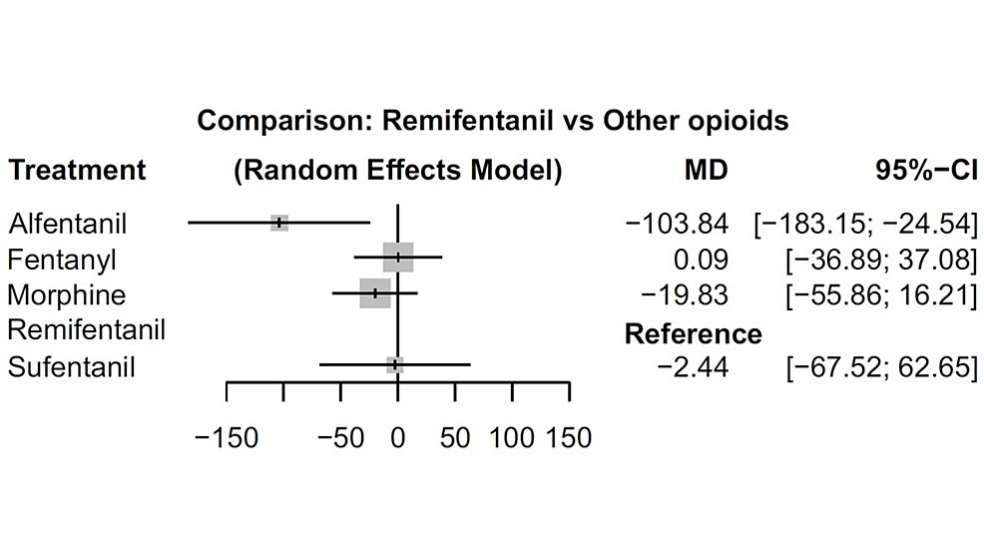
Forest plots for the association of remifentanil and other opioids with primary outcomes (duration of mechanical ventilation). Outcomes are reported as the mean difference (MD) with 95% confidence intervals (CIs).

**Figure 4:**
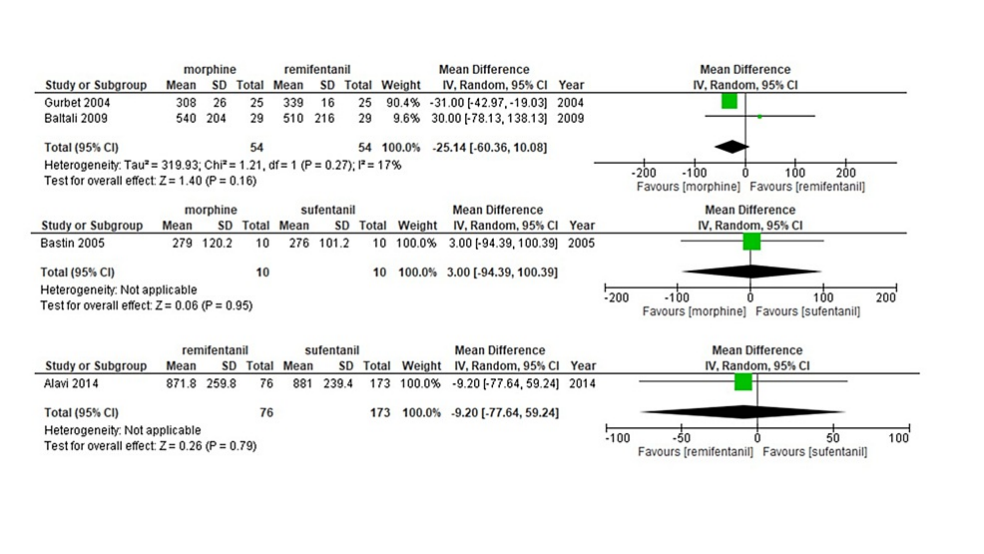
Forest plot of each comparison for length of mechanical ventilation Outcomes are reported as the mean difference (MD) with 95% confidence intervals (CIs).

All eight studies were included in the analysis of MV duration. Neither fentanyl (mean difference [MD] 0.09 min; 95%CI -36.89-37.08; moderate confidence evidence) nor morphine (MD; -19.83 min; 95%CI -55.86-16.21; moderate confidence evidence) or sufentanil (MD; -2.44 min; 95%CI -67.52-62.65; low confidence evidence) administration correlated with longer durations of MV compared to remifentanil (Figure 3, Figure 4, Table 4, and Table 5). Our data also suggest that remifentanil prolongs the MV time compared to alfentanil (mean difference [MD] -103.84 min; 95%CI -183.15-24.54; moderate confidence evidence).

**Table 4:** Summary of confidence in the results of a network meta-analysis for the duration of mechanical ventilation

Comparison	Number of studies	Within-study bias	Reporting bias	Indirectness	Imprecision	Heterogeneity	Incoherence	Confidence rating
Alfentanil vs Morphine	2	No concerns	Low risk	No concerns	No concerns	Some concerns	No concerns	Moderate
Fentanyl vs Morphine	2	No concerns	Low risk	No concerns	No concerns	Some concerns	No concerns P-0.881＊	Moderate
Fentanyl vs Remifentanil	2	No concerns	Low risk	No concerns	No concerns	Major concerns	No concerns P=0.283＊	Moderate
Morphine vs Remifentanil	2	No concerns	Low risk	No concerns	No concerns	Some concerns	No concerns P=0.555＊	Moderate
Morphine vs Sufentanil	1	No concerns	Low risk	No concerns	No concerns	Major concerns	No concerns P=0.497＊	Moderate
Remifentanil vs Sufentanil	1	Major concerns	Low risk	No concerns	Some concerns	Some concerns	No concerns P=0.497＊	Low
Alfentanil vs Fentanyl	0	No concerns	Low risk	No concerns	No concerns	Some concerns	No concerns	Moderate
Alfentanil vs Remifentanil	0	No concerns	Low risk	No concerns	No concerns	Some concerns	No concerns	Moderate
Alfentanil vs Sufentanil	0	No concerns	Low risk	No concerns	No concerns	Some concerns	No concerns	Moderate
Fentanyl vs Sufentanil	0	Major concerns	Low risk	No concerns	Some concerns	Some concerns	No concerns	Low
＊ We used a side-splitting approach as a local method								

**Table 5:** League table of duration of mechanical ventilation

Opioid	Alfentanil	Morphine	Sufentanil	Remifentanil	Fentanyl
Alfentanil	-	83.20 (-1237.64 to 1603.80)	201.37 (-1288.77 to 2775.61)	103.84 (24.54 to 183.15)	372.29 (-924.52 to 1997.13)
Morphine	-83.20 (-1603.80 to 1237.64)	-	108.22 (-1005.12 to 1809.83)	-19.83 (-55.86 to16.21)	293.70 (-176.96 to 816.94)
Sufentanil	-201.37 (-2775.61 to 1288.77)	-108.22 (-1809.83 to 1005.12)	-	-2.44 ( -67.52 to 62.65)	173.70 (-1515.83 to 1335.23)
Remifentanil	--103.84 (-183.15 to -24.54)	19.83 (-16.21 to 55.86)	2.44( -62.65 to 67.52)	-	0.09 ( -36.89 to 37.08)
Fentanyl	-372.29 (-1997.13 to 924.52)	-293.70 (-816.94 to 176.96)	-173.70 (-1335.23 to 1515.83)	-0.09 ( -37.08 to 36.89)	-
Outcomes are reported as mean difference (MD) with 95% confidence intervals (CIs).					

Secondary Outcomes

**Figure 5:**
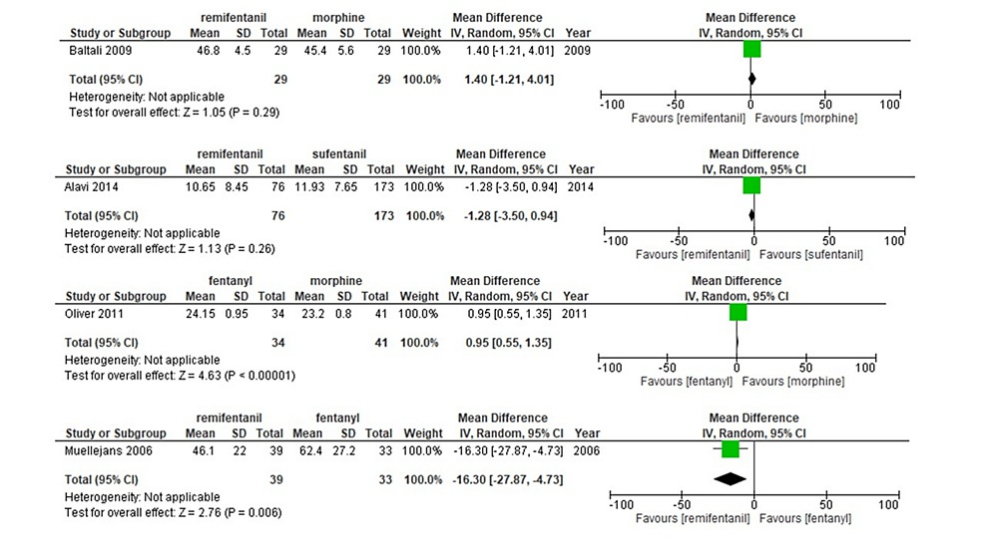
Forest plot of each comparison for the duration of ICU stay Outcomes are reported as the mean difference (MD) with 95% confidence intervals (CIs).

ICU length of stay: Four of the eight studies reported the ICU length of stay [14-16,22]. As the number of studies was too small, the NMA could not be conducted. A meta-analysis was also not possible, because there was only one comparison of remifentanil with each other opioid. Remifentanil was associated with shorter ICU stays than fentanyl but resulted in prolonged ICU stays compared to morphine. There was no significant difference in the length of ICU stay between the remifentanil and the sufentanil or alfentanil group (Table 6) (Figure 5).

**Table 6:** Summary of the risk of bias for the length of ICU stay

Authors	Bias arising from the randomization process	Bias due to deviations from intended interventions	Bias due to missing outcome data	Bias in measurement of the outcome	Bias in selection of the reported result	Overall risk of bias
Muellejans 2006 [17]	Low Risk	Low Risk	﻿High Riska	Low Risk	Some Concerns	High Risk
Baltali 2009 [16]	Low Risk	Some Concerns	Low Risk	Low Risk	Some Concerns	Some Concerns
Oliver 2011 [15]	Low Risk	Low Risk	Low Risk	Low Risk	Some Concerns	Some Concerns
Alavi 2014 [14]	High Riskb	Some Concerns	Low Risk	Low Risk	Some Concerns	High Risk
a. Outcome's are missing 10%. b. No description of allocation method						

**Figure 6:**
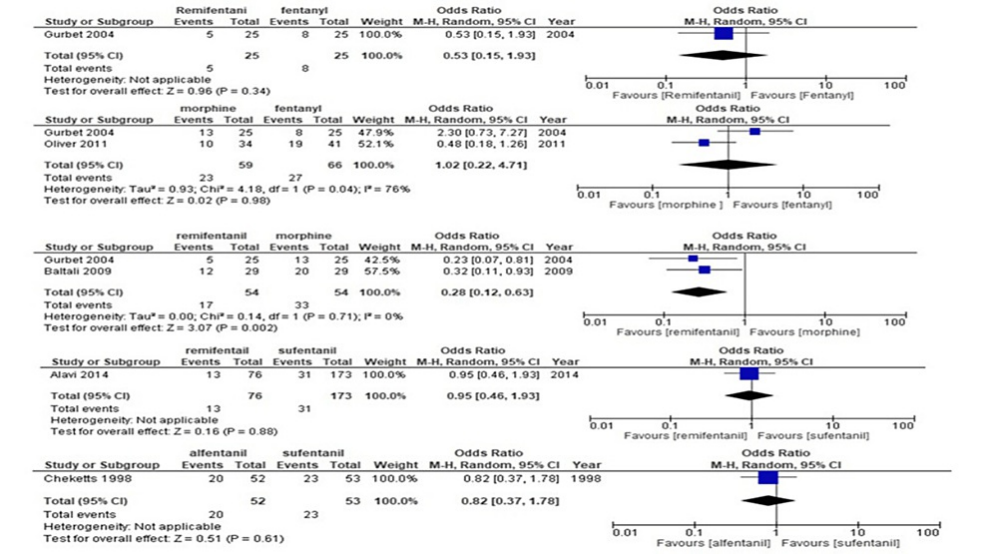
Forest plot of each comparison for vomiting/nausea The outcome was shown with confidence interval (CI) and Mantel-Haenszel (M-H)

Vomiting/nausea: One study compared remifentanil to fentanyl [19] regarding the occurrence of vomiting/nausea, two studies compared remifentanil to morphine [15,19], and one study compared remifentanil to sufentanil [14]. The number of studies was too small to perform the NMA; therefore, the results are presented pairwise. Our findings show that remifentanil induces fewer episodes of vomiting/nausea than morphine (odds ratio [OR], 0.28; 95%CI, [0.12-0.63]) (Table 7) (Figure 6).

**Table 7:** Summary of risk of bias for vomiting/nausea

Authors	Bias arising from the randomization process	Bias due to deviations from intended interventions	Bias due to missing outcome data	Bias in measurement of the outcome	Bias in selection of the reported result	Overall risk of bias
Cheketts 1998	Low Risk	Low Risk	Low Risk	Low Risk	Some Concerns	Some Concerns
Gurbet 2004	Some Concerns	Low Risk	Low Risk	Low Risk	Some Concerns	Some Concerns
Baltali 2009	Low Risk	Some Concerns	Low Risk	Low Risk	Some Concerns	Some Concerns
Oliver 2011	Low Risk	Low Risk	Low Risk	Low Risk	Some Concerns	Some Concerns
Alavi 2014	High Risk^a^	Some Concerns	Low Risk	Low Risk	Some Concerns	High Risk
a. No description of allocation method						

**Figure 7:**
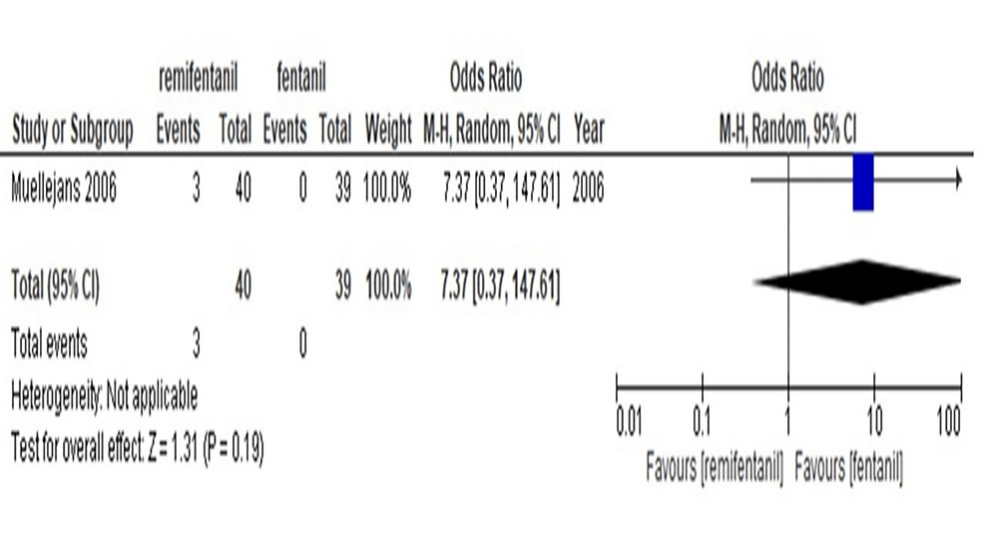
Forest plot of each comparison for hemodynamic instability The outcome was shown with confidence interval(CI) and Mantel-Haenszel (M-H)

Hemodynamic instability: Only one study compared hemodynamic instability between patients administered remifentanil and those treated with fentanyl [17]. As the number of studies was too small to perform the NMA, the results are presented pairwise. Table 8 and Figure 7 show that remifentanil has a greater tendency for hemodynamic instability than fentanyl (OR, 7.37; 95%CI, [0.37-0.147.61]).

**Table 8:** Summary of the risk of bias for hemodynamic instability

Authors	Bias arising from the randomization process	Bias due to deviations from intended interventions	Bias due to missing outcome data	Bias in measurement of the outcome	Bias in selection of the reported result	Overall risk of bias
Muellejans 2006 [17]	Low Risk	Low Risk	﻿High Risk^a^	Low Risk	Some Concerns	High Risk
a. Outcome's are missing 10%.						

**Figure 8:**
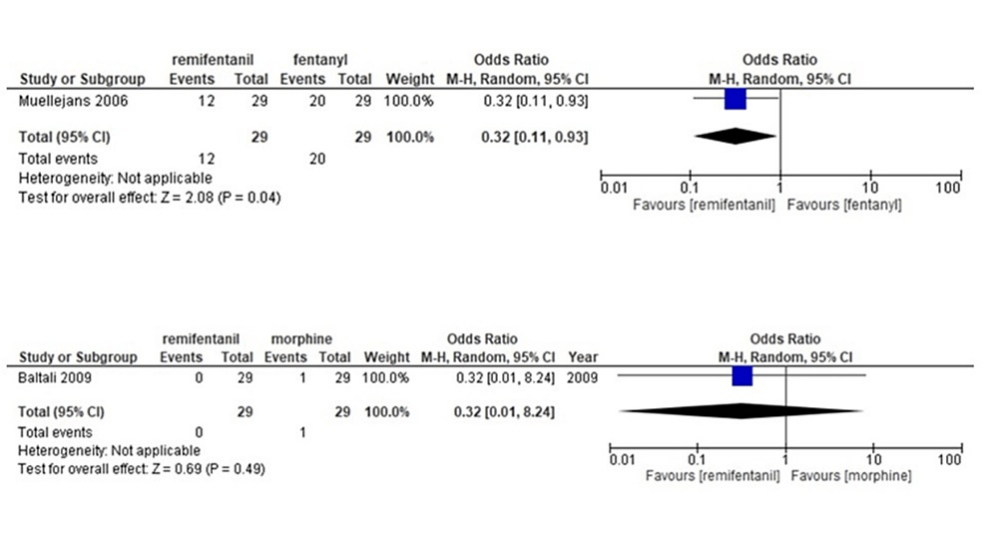
Forest plot of each comparison for delirium The outcome was shown with confidence interval (CI) and Mantel-Haenszel (M-H)

Delirium: One study assessed the occurrence of delirium in patient groups administered remifentanil or morphine [16], and one in groups treated with remifentanil or fentanyl [17]. The number of studies was too small to perform the NMA, and the results are therefore presented pairwise. Our findings show that remifentanil use reduces the incidence of delirium compared with fentanyl use (OR, 0.32; 95%CI, [0.11-0.93]), but not as much as morphine (OR, 0.32; 95%CI, [0.01-8.24]) (Table 9) (Figure 8).

**Table 9:** Summary of the risk of bias for delirium

Authors	Bias arising from the randomization process	Bias due to deviations from intended interventions	Bias due to missing outcome data	Bias in measurement of the outcome	Bias in selection of the reported result	Overall risk of bias
Muellejans 2006	Low Risk	Low Risk	﻿High Risk^a^	Low Risk	Some Concerns	High Risk
Baltali 2009	Low Risk	Some Concerns	Low Risk	Low Risk	Some Concerns	Some Concerns
a. Outcome's are missing 10%.						

Subgroup Analysis

**Figure 9:**
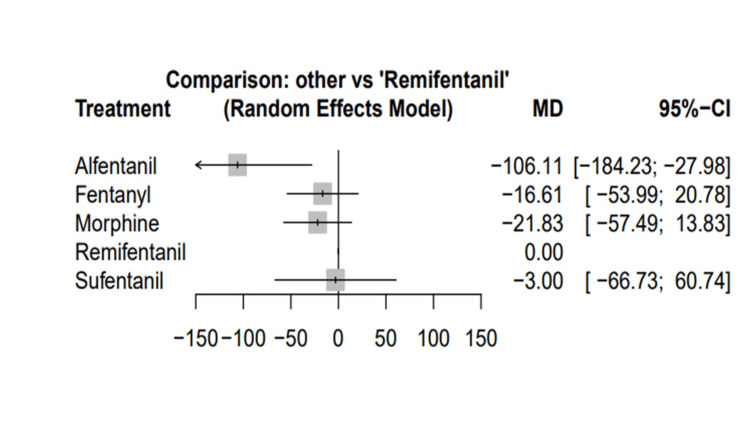
Forest plot of the subgroup analysis for studies with sedative medications Outcomes are reported as the mean difference (MD) with 95% confidence intervals (CIs).

We performed a subgroup analysis of six studies, excluding two that used concomitant sedative medications [14,16,18-21]. We found that remifentanil does not reduce MV time compared to morphine, fentanyl, or sufentanil; it also seems to prolong the MV time compared to alfentanil (Appendix 3 Figure 9).

Sensitivity Analysis

**Figure 10:**
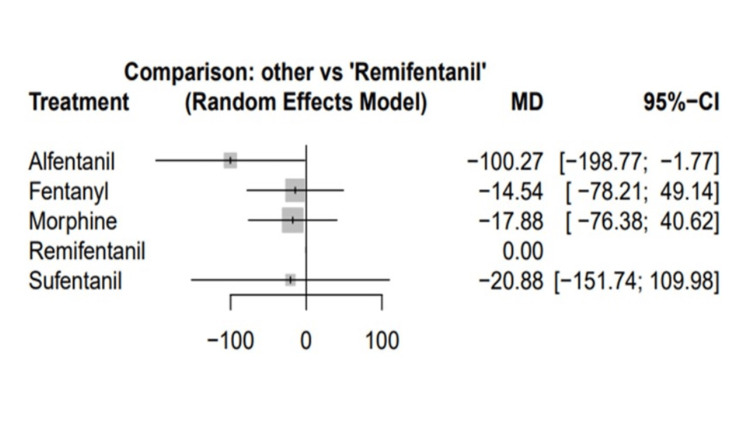
Forest plot of the sensitivity analysis for excluding studies with a high risk of bias. Outcomes are reported as the mean difference (MD) with 95% confidence intervals (CIs).

**Figure 11:**
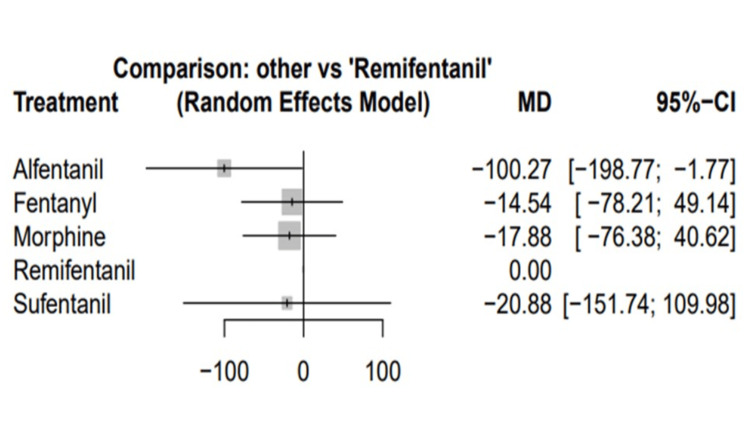
Forest plot of the sensitivity analysis for excluding studies with missing data. Outcomes are reported as the mean difference (MD) with 95% confidence intervals (CIs).

**Figure 12:**
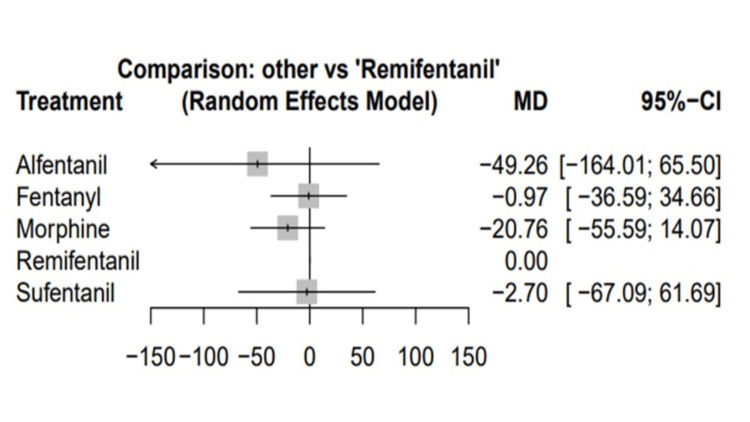
Forest plot of the sensitivity analysis for excluding studies where the outcome is time to extubation Outcomes are reported as the mean difference (MD) with 95% confidence intervals (CIs).

We performed a sensitivity analysis on five studies, excluding three with a high risk of bias [16,18-21]. We also performed an additional sensitivity analysis on six studies, excluding two with missing data [14,16,18-21]. These results reveal that remifentanil administration does not reduce MV time compared to other opioids. They also suggest that remifentanil prolongs the MV time compared to alfentanil. In addition, a sensitivity analysis on seven studies [14-19,21] was performed excluding studies in which the outcome was time to extubation. Results showed that remifentanil prolonged ventilatory time compared to other opioids (Appendix 4, Figure 10, Figure 11, Figure 12).

Discussion

Interpretation of Findings

Our systematic review encompassing eight RCTs with a total of 691 participants indicates that remifentanil does not reduce the duration of MV compared to other commonly used opioids (fentanyl, morphine, or sufentanil) in the ICU setting after cardiac surgery. This is somewhat surprising, given the ultra-short duration of action of remifentanil, which has been postulated to offer advantages in reducing MV times.

Context of Previous Studies

Three existing systematic reviews have provided valuable but inconsistent and highly heterogeneous findings regarding the efficacy of remifentanil compared with other opioids in patients undergoing MV [4-6]. Tan et al. (2009) [4] and Zhu et al. (2017) [5] both conducted meta-analyses comparing remifentanil with other opioids and sedatives; while Tan et al. [4] found no significant benefits in terms of ventilation duration, mortality, or ICU stay, they noted that remifentanil may shorten the intubation time after sedation. Zhu et al. [5] reported inconclusive results regarding the efficacy of remifentanil, with high data heterogeneity (I2 = 89%); a subgroup analysis comparing remifentanil with fentanyl, morphine, and sufentanil also revealed a high level of heterogeneity, necessitating further research. In contrast, Yang et al. [6] conducted a systematic review focusing on critically ill adult patients and included only RCTs that compared the analgesic effects of remifentanil with those of other opioids. These authors found that remifentanil was associated with a reduction in the duration of MV compared with other opioids (SMD -0.23; 95%CI -0.41--0.06; P=.01; IV random; heterogeneity I2= 50%, P=.01). The results reported by Yang et al. [6] differed from our results because of the difference in the studies included.

Specifically, the study by Dahaba et al. in 2004 [23] (N=40; remifentanil vs. morphine) and the study by Le Guen et al. in 2013 [24] (N=60; remifentanil vs. fentanyl) concluded that remifentanil reduces the duration of MV; both of these studies focused on postoperative patients.

Our NMA, which included eight RCTs involving 691 ICU patients after cardiac surgery, did not support the superiority of remifentanil in reducing MV times compared with other commonly used opioids, in contrast with Yang et al. [6], who suggested that remifentanil is associated with shorter durations of MV. This discrepancy likely stems from variations in the included studies, highlighting the fact that the role of remifentanil in ICU pain management has not yet been firmly established and warrants further investigation.

A subgroup analysis specific to cardiac surgery patients suggested that remifentanil could reduce the time on a ventilator by an average of 0.51 hours compared to other opioids (95%CI -1.46-0.44). This finding aligns with the most recent medical guidelines [7], which advocate the postsurgical use of remifentanil. It is important to note, however, that our study included data from the 2004 study by Gurbet et al. [19], one that was not considered in some previous analyses. We applied NMA, which enabled us to assess studies with multiple comparison groups. The inclusion of the study by Gurbet and colleagues [19], which evaluated the efficacy of remifentanil against fentanyl and morphine, revealed that remifentanil in fact extended the duration of MV compared to these other opioids. Additionally, several studies [25-28] were considered in previous analyses but not in ours. They were excluded from our review because they primarily focused on opioid use during cardiac surgery, whereas we explicitly examined the postoperative period. The selective focus of our analysis thus contributes to the variance between our findings and those of previous studies; however, the debate over the inclusion or exclusion of interventions during cardiac surgery remains unresolved, partly because the pool of relevant studies is relatively small overall. Moreover, the availability of different types of opioids varies by country, which complicates international comparisons. Future research should aim to compare the individual use of remifentanil against specific opioids, rather than lumping all other opioids together for a general comparison. Another influencing factor could be the mean duration of MV in the studies included in our analysis, which was approximately 500 min. This duration may have been too short to detect any significant advantage of remifentanil over other opioids in terms of reducing ventilation time. However, the available research is insufficient to confirm this hypothesis.

Regarding side effects, our study suggests that remifentanil has the advantage of reducing incidences of nausea and vomiting compared to morphine but also indicates greater hemodynamic instability compared to fentanyl. Furthermore, our data indicate that remifentanil may reduce occurrences of delirium compared to fentanyl. Prior studies have found no significant differences between remifentanil and other opioids in terms of nausea and vomiting, hypotension, agitation, and delirium [5,6]. In addition, opioid-induced hyperalgesia, the most characteristic AE of remifentanil, was not observed in any of the studies included in this review. Opioid-induced hyperalgesia has been reported in postoperative patients [29], however, and the lack of studies on this side effect is an important issue.

Implications for the Clinical Practice and Future Research

Our systematic review demonstrates that remifentanil does not confer an advantage in reducing the duration of mechanical ventilation compared to other commonly used opioids, such as fentanyl, morphine, and sufentanil. The safety profile of remifentanil also requires careful consideration; while it appears to be more hemodynamically unstable than fentanyl, it seems to have fewer gastrointestinal side effects, such as vomiting or nausea, than morphine. Some studies have reported intraoperative and postoperative interventions for postoperative cardiac opioid use, but few have compared them to the administration of remifentanil. In Japan, remifentanil and fentanyl are the two main opioids used in the postoperative ICU, and clinical studies have been conducted on their use. We believe that a comparison of remifentanil and fentanyl in patients undergoing cardiac surgery in Japan using a unified technique and intraoperative sedatives and analgesics is necessary. In addition, regarding side effects, the fact that opioid-induced hyperalgesia specific to remifentanil has not been investigated in previous studies suggests that this is an area in need of further research.

Limitations

Our study has several limitations that should be addressed in future research. First, we did not consider the effect of general anesthetic agents as a confounding variable when assessing patients after cardiac surgery. This omission might have skewed our findings related to the duration of MV. Second, in our study, it was treated as equivalent to the duration of mechanical ventilation. The results of the sensitivity analysis did not affect the directionality of each drug, suggesting that the results of this integrated study remain unchanged. Third, there is a dearth of studies that directly compare remifentanil to other agents, particularly alfentanil. The evidence we present concerning alfentanil is based on indirect comparisons, highlighting the need for future studies to perform direct comparisons. Fourth, our research mainly focused on patients who underwent cardiac bypass surgery, neglecting the diversity of outcomes that may be relevant to other types of cardiac procedures. We were unable to disaggregate the data based on the type of surgery performed, which is another area for further investigation. Overall, these limitations underscore the need for more comprehensive and targeted studies to provide clearer insights into the role of remifentanil in postcardiac surgery settings.

## Conclusions

RCTs that compared various opioids in adults admitted to the intensive care unit after cardiac surgery were identified. Our NMA revealed no benefits of remifentanil compared with other commonly used opioids, such as fentanyl, morphine, or sufentanil, in shortening the duration of MV in ICU patients after cardiac surgery.

## Appendices

Additional file 1. PRISMA NMA Checklist of Items to Include When Reporting A Systematic Review Involving a Network Meta-analysis. We followed the Preferred Reporting Items for SR and Meta-Analysis 2020 (PRISMA-2020) for preparing this protocol.(https://osf.io/yahw2/)

Additional file 2. Search strategy

MEDLINE (PubMed) search strategy (Appendix 1)

#1: "Cardiac Surgical Procedures"[MeSH]

#2: "Cardiac surgery"[tiab]

#3 #1 or #2

#4 "Remifentanil"[MeSH]

#5 "Remifentanil"[tiab]

#6 "Fentanyl"[MeSH]

#7 "Fentanyl"[tiab]

#8 "Morphine"[MeSH]

#9 "Morphine"[tiab]

#10 "Sufentanil"[MeSH]

#11 "Sufentanil"[tiab]

#12 "Alfentanil"[MeSH]

#13 "Alfentanil"[tiab]

#14 "Analgesics, Opioid"[MeSH]

#15 #4 or #5 or #6 or #7 or #8 or #9 or #10 or #11 or #12 or #13 or #14

#16 #3 and #15

#17 ("Randomized Controlled Trial"[pt] OR "Controlled Clinical Trial"[pt] OR "Clinical Trials as Topic"[mh] OR randomized[tiab] OR placebo[tiab] OR randomly[tiab] OR trial[tiab] OR groups[tiab]) NOT (Animals [mh] NOT Humans [mh])

#18 #16 and #17

CENTRAL (Cochrane Library) search strategy (Appendix2)

#1: MeSH descriptor: [Cardiac Surgical Procedures] explode all trees

#2: (Cardiac Surgical Procedures):ti,ab,kw

#3: #1 or #2

#4 MeSH descriptor: [Remifentanil] explode all trees

#5 (Remifentanil):ti,ab,kw

#6 MeSH descriptor: [Fentanyl] explode all trees

#7 (Fentanyl):ti,ab,kw

#8 MeSH descriptor: [Morphine] explode all trees

#9 (Morphine):ti,ab,kw

#10 MeSH descriptor: [Sufentanil] explode all trees

#11 (Sufentanil):ti,ab,kw

#12 MeSH descriptor: [Alfentanil] explode all trees

#13 MeSH descriptor: [Analgesics, Opioid] explode all trees

#14 #3 and #13

#15 #4 or #5 or #6 or #7 or #8 or #9 or #10 or #11 or #12 or #13 or #14

#16 #3 and #15

 EMBASE (Dialog) search strategy (Appendix3)

S1 (EMB.EXACT.EXPLODE("heart surgery")) S2 ab(Cardiac surgery) OR ti(Cardiac surgery) S3 S2 OR S1 S4 EMB.EXACT.EXPLODE("remifentanil") S5 (ab(remifentanil) OR ti(remifentanil)) S6 EMB.EXACT.EXPLODE("fentanyl") S7 (ab(Fentanyl) OR ti(Fentanyl)) S8 EMB.EXACT.EXPLODE("morphine") S9 (ab(Morphine) OR ti(Morphine)) S10 EMB.EXACT.EXPLODE("sufentanil") S11 (ab(Sufentanil) OR ti(Sufentanil)) S12 EMB.EXACT.EXPLODE("alfentanil") S13 (ab(Alfentanil) OR ti(Alfentanil)) S14 (EMB.EXACT.EXPLODE("narcotic analgesic agent")) S15 S14 OR S13 OR S12 OR S11 OR S10 OR S9 OR S8 OR S7 OR S6 OR S5 OR S4 S16 S15 AND S3

S17 ((EMB.EXACT("double blind procedure")) OR (ab(double NEAR/1 blind*) OR ti(double NEAR/1 blind*)) OR (ab(placebo*) OR ti(placebo*)) OR (ab(blind*) OR ti(blind*)))

S18 S16 AND S17

ICTRP search strategy (Appendix4)

(remifentanil or fentanyl or morphine or sufentanil or alfentanil) AND (“cardiac surgery”)

ClinicalTrials.gov search strategy (Appendix5)

Condition or disease: “cardiac surgery”

Intervention: opioid OR remifentanil OR fentanyl OR morphine OR sufentanil OR alfentanil

Additional file 3. Subgroup analysis

Additional file 4. Sensitivity analysis
